# Optic Nerve Head and Retinal Changes in Idiopathic Intracranial Hypertension: Correlation with Short-Term Cerebrospinal Fluid Pressure Monitoring

**DOI:** 10.3390/jcm13020562

**Published:** 2024-01-18

**Authors:** Mario Damiano Toro, Niccolò Castellino, Andrea Russo, Davide Scollo, Teresio Avitabile, Robert Rejdak, Magdalena Rejdak, Vincenzo Cimino, Ciro Costagliola, Adriano Carnevali, Clara Grazia Chisari

**Affiliations:** 1 Eye Clinic, Public Health Department, University of Naples Federico II, 80131 Naples, Italy; 2 Chair and Department of General and Pediatric Ophthalmology, Medical University of Lublin, 20-079 Lublin, Poland; 3 Department of Ophthalmology, University of Catania, 95123 Catania, Italy; 4 Department of Ophthalmology, University Hospital of Zurich, 8091 Zurich, Switzerland; 5 Spinal Cord Unit, Cannizzaro Hospital, 95100 Catania, Italy; 6 Department of Neurosciences, Reproductive Sciences and Dentistry, University of Naples Federico II, 80138 Naples, Italy; 7 Department of Ophthalmology, University Magna Grecia of Catanzaro, 88100 Catanzaro, Italy; 8 Department “GF Ingrassia”, Section of Neurosciences, University of Catania, 95125 Catania, Italy

**Keywords:** optical coherence tomography, retina, idiopathic intracranial hypertension, macula, optic nerve abnormalities

## Abstract

Background: We aimed to assess the status of the optic nerve and retina by optical coherence tomography (OCT) in a group of patients with idiopathic intracranial hypertension (IIH) on the basis of dynamic changes in intracranial pressure. Methods: This observational and cross-sectional study included patients affected by idiopathic intracranial hypertension with papilledema (IIHWP) and patients with idiopathic intracranial hypertension without papilledema (IIHWOP). All participants underwent an OCT examination of the macula and optic nerve head. Parameters related to intracranial pressure, including cerebrospinal fluid (CSF) opening pressure (oCSFp), CSF mean pressure (mCSFp), and pulse wave amplitude (PWA), were included in the analysis. Results: Out of the 22 subjects enlisted for the study, a total of 16 patients suggestive of IIH were finally enrolled. Papilledema was detected in nine subjects (56.2%) and seven patients were affected by IIHWOP (43.7%). The OCT examination showed a higher mean RNFL thickness in IIHWP patients in comparison to IIHWOP in both eyes (*p* < 0.05 and *p* < 0.01, respectively). Intracranial pressure (ICP) measurements showed that IIHWP had higher values of oCSFp, mCSFp, and PWA compared to IIHWOP (*p* = 0.0001, *p* = 0.0001, and *p* = 0.0001, respectively). In addition, ICP parameters significantly correlated with RNFL. Conclusions: Clinical parameters suggestive of idiopathic intracranial hypertension are associated with retina and optic nerve OCT parameters. OCT is a useful tool to detect these alterations in a non-invasive fashion.

## 1. Introduction

Idiopathic intracranial hypertension (IIH) syndrome is characterized by elevated intracranial pressure (ICP) in the absence of a mass lesion, venous sinus thrombosis, or hydrocephalus [1,2]. IIH typically presents with several symptoms, including high-frequency headaches, transient visual obscuration, pulsatile tinnitus, and blurred or double vision. IIH is usually observed in young women of childbearing age [3,4], and its frequency reflects the increased prevalence of obesity [5]. Magnetic resonance venography (MRV) usually depicts bilateral cerebral transverse venous sinus stenosis [6,7]. Papilledema is a common sign and, if not treated, can result in insidious and slowly progressive visual loss, leading to the development of retinal atrophy and permanent visual impairment [8,9]. Additionally, IIH without papilledema (IIHWOP) has been identified as a variant of the form with papilledema (IIHWP) [10,11,12,13,14]. According to recent studies, IIH with and without papilledema share similar features, including refractory headaches and radiological signs such as cerebral transverse venous sinus stenosis [15], but the reason why some IIH patients develop papilledema, while others do not, remains controversial. It has been proposed that the development of IIHWOP could be explained by (1) anatomical variants in the optic nerve sheaths or in its trabeculations protecting from elevated ICP in the optic disc (OP); (2) the fluctuation in ICP values below the threshold responsible for papilledema; (3) considering IIHWOP as a presymptomatic form of IIH which improves or disappears prior to developing papilledema [13,16].

It is known that a chronic elevation in ICP leads to several biomechanical alterations in the optic nerve head (ONH) [17,18,19,20]. Some studies have demonstrated that retinal and ONH parameters, measured by optical coherence tomography (OCT), are correlated with increased ICP in papilledema due to IIH [17,21,22,23]. OCT is a widely employed imaging technique that allows us to assess neuroretinal changes in a non-invasive fashion.

According to several studies, the lumbar subarachnoid space and ONH regions are directly connected and, thus, OCT may represent a suitable non-invasive tool for monitoring ICP and for determining objective changes in the ONH after an acute decrease in lumbar cerebrospinal fluid (CSF) pressure [20,24]. Even though the literature reporting the effects of ICP on diseases causing papilledema is rapidly growing, research on the effects of dynamic ICP on optic nerve structures in IIHWP and IIHWOP is lacking. 

In order to better understand the impact of papilledema, we segregated patients with papilledema and without papilledema into distinct groups. Thus, we designed an observational and cross-sectional study to investigate the status of the optic nerve head and retina in patients affected by IIH using OCT and the association with parameters suggestive of ICP.

## 2. Materials and Methods

This observational and cross-sectional study screened patients with high-frequency or daily persistent headaches, consecutively admitted to the Department of General Ophthalmology of the Medical University of Lublin (Lublin, Poland) from December 2020 to December 2021. The study was conducted in accordance with the Declaration of Helsinki and was approved by the Institutional Review Board of the Medical University of Lublin (ethical committee number KE-0254/123/2020). Overall, 22 patients were selected for the study, including those with clinical and radiological evidence suggestive of IIH with or without papilledema. All patients underwent brain MRI and MRI-venography (MRV). 

### 2.1. Inclusion Criteria

1. Clinical evidence suggestive of IIH with or without papilledema:
  - High-frequency headache attacks persisting for at least one year refractory to prophylactic therapy;
  - High-frequency headache attacks with papilledema, or transient or persistent visual obscuration, and/or blurred vision, or diplopia.
2. Normal neurological examination.
3. Evidence of bilateral stenosis of the cerebral transverse sinus at MRV [25].

### 2.2. Exclusion Criteria

1. Evidence of central nervous system disease at brain MRI.
2. Evidence of current or previous cerebral venous thrombosis revealed by cerebral MRV.
3. Treatment with psychoactive, antihypertensive, cardiac, or other drugs interfering with the CSF pressure.
4. Comorbidities such as diabetes mellitus, hypertension, cardiovascular and renal diseases, neurological diseases (i.e., tumors, ictus, infectious diseases, epilepsy, multiple sclerosis, previous head or eye injury).
5. Eyes with optic disc and retinal pathology (including anterior ischemic optic neuropathy, posterior uveitis, and central retinal vein occlusion), which could bias measurements, and eyes affected by corneal opacity or cataract for which OCT imaging could not be obtained were ruled out from the analysis.

Approval from the local committee for the study protocol and consent for participation from the enrolled patients were both obtained before commencing the study.

Lumbar puncture and short CSF dynamic monitoring were performed to evaluate those patients with evidence of cerebral venous outflow disturbances at MRV documented by the presence of bilateral cerebral transverse sinus stenosis. The ophthalmological evaluation and the OCT were performed in all enrolled patients, 24 h before the ICP measurement.

Ophthalmological evaluation: all the participants were subjected to a comprehensive ophthalmological assessment, including best corrected visual acuity (BCVA) testing with slit lamp biomicroscopy, dilated fundus examination, Snellen charts, and intraocular pressure measurement by applanation tonometry. 

**Figure 1:**
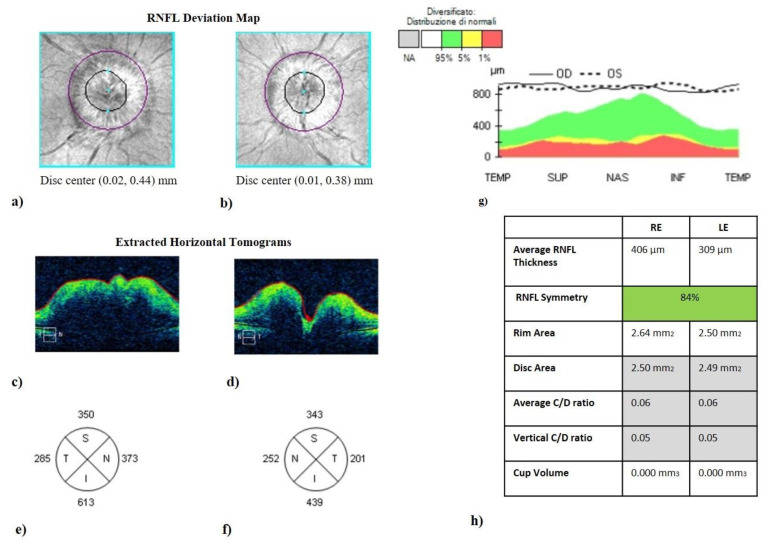
Optic disc cube (200 × 200 protocol) centered on the optic nerve head (ONH). It represents the optical coherence tomography of a patient with idiopathic intracranial hypertension without papilledema (IIHWP), demonstrating the presence of bilateral papilledema, more prominent in the right eye. (**a**,**b**) Images of ONH (bordered by a dark line). ONH swelling and consequent increased thickness in the right eye (**c**) and in the left eye (**d**) are discernible looking at horizontal B-scans. Thickening of RNFL of all quadrants can be observed in (**e**–**g**). An overview of OCT parameters is represented in (**h**). IIHWP: idiopathic intracranial hypertension with papilledema; OCT: optical coherence tomography; ONH: optic nerve head; RNFL: retinal nerve fiber layer; RE: right eye; LE: left eye.

OCT imaging: The Stratus OCT (Cirrus OCT 5000, Carl Zeiss Meditec, Dublin, CA, USA) was used to perform the OCT. A single operator masked to the patients’ diagnosis recorded an average of three sequential circular scans of diameter 3.46 mm centered on the optic disc. All scans were collected after pupil dilation with 1% tropicamide. The optic disc cube 200 × 200 protocol for the ONH (Figure 1) and the Macular Cube 512 × 128 protocol centered on the fovea were carried out in order to obtain peripapillary and macular data, respectively. Scans with a signal strength less than 7/10 were not considered for further data analyses. For each eye, we analyzed the mean retinal nerve fiber layer (RNFL) thickness and single-quadrant thickness, both calculated automatically by OCT using the existing software. We also evaluated the ganglion cell layer (GCL), foveal thickness (FT), and macular volume (MV) of all the patients. Neuroretinal rim area (NRRA, µm^2^) and thickness (NRRT, µm), cup volume (CV, µm^3^), and cup-to-disc ratio (C/D ratio) were also calculated automatically. The OCT automated segmentation was validated by two independent expert OCT examiners (T.M.D. and R.M.). Quality control and APOSTEL guidelines according to published criteria were applied [26,27,28].

ICP monitoring: The monitoring was performed for 1 h in a silent room. After a pre-treatment with local anesthesia (subcutaneous lidocaine), the lumbar puncture was conducted at the L3–L4 level in the lateral decubitus position using a Quincke 20-gauge needle with a three-way stopcock. We measured the CSF opening pressure (oCSFp), CSF mean pressure (mCSFp), and pulse wave amplitude (PWA). IIH was diagnosed when the CSF opening pressure or CSF mean pressure was >250 mmH_2_O confirmed by lumbar ICP monitoring [25]. 

The recording of oCSFp was commenced four minutes after the CSF pressure monitoring started (the opening pressure phase) [29]. Both the mCSFp and the CSF mean PWA were recorded from the culmination of the opening phase to the sixteenth minute (short-term monitoring). We also calculated the CSF PWA as the peak-to-peak amplitude, where the systolic peak was the highest and the diastolic peak of the CSF pressure was the lowest. The upper normal limit of CSF PWA was 54.8 mmH_2_O [30,31]. 

We excluded from the analysis the values of CSF pressure and PWA collected during artifact period (i.e., movements of the arms or of the head, coughing, etc.).

Statistical analysis: The normality of all the values included in the analysis was assessed using the Shapiro–Wilk test. The differences between subgroups were evaluated with Student’s *t*-tests and Mann–Whitney tests for parametric and for non-parametric variables, respectively. For correlation analysis, the Pearson correlation coefficient (r) was used. Statistical significance was indicated by a probability value (*p*) of 0.05. STATA 11 was used for all statistical analyses (Boston RC, Sumner AE. 2003) [32].

## 3. Results

Out of the 22 subjects recruited for the study, a total of 16 patients suggestive of IIH were finally enrolled. Six patients were excluded on the basis of ICP lumbar monitoring, which resulted in the range of normal limits. None of the selected patients presented systemic hypertension at the moment of the ICP monitoring. Only three patients abused symptomatic treatment (two patients of triptans and one of non-steroid anti-inflammatory drugs). All selected patients were divided into two groups according to the finding of papilledema: nine IIHWP (56.2%) and seven IIHWOP (43.7%). Clinical and demographical data are shown in Table 1. Visual acuity (VA) was markedly reduced in patients with papilledema.

ICP measurement showed that IIHWP had higher values of oCSFp, mCSFp, and PWA compared to IIHWOP (Table 2). The OCT examination showed that a higher mean RNFL thickness in IIHWP patients was detected in comparison to IIHWOP in both eyes (209.4 vs. 98.5, *p* < 0.05; 166.6 vs. 98.6, *p* < 0.01 in right- and left-eye groups, respectively). IIHWP showed lower values of CV and C/D ratio compared to IIHWOP; the complete data are reported in Table 3. 

**Figure 2:**
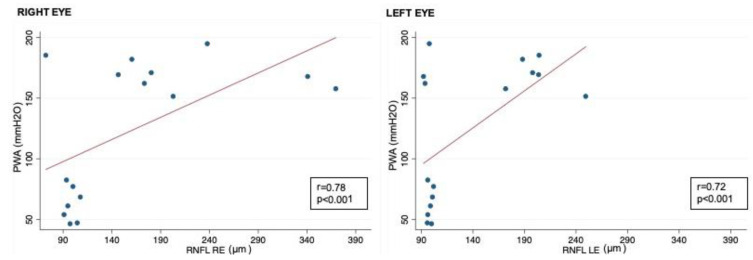
Correlation between CSF PWA and RNFL in right and left eyes; RNFL: retinal nerve fiber layer; CSF PWA: cerebrospinal fluid pressure pulse wave amplitude; r: Spearman correlation coefficient.

**Figure 3:**
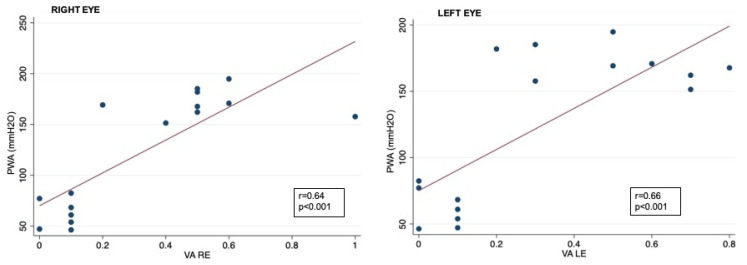
Correlation between CSF PWA and visual acuity data in right and left eyes; VA: visual acuity expressed as logarithm of minimal angle of resolution (LogMAR); CSF PWA: cerebrospinal fluid pressure pulse wave amplitude; r: Spearman correlation coefficient.

Correlation analysis showed that RNFL thickness was significantly associated with oCSFp, mCSFp, and PWA; in contrast, C/DR and CV inversely correlated with oCSFp, mCSFp, and PWA (Table 4, Figure 2). We also found a positive correlation between rim thickness and both mCSFp and PWA, but not oCSFp (Table 4). VA values showed a significant correlation with OCT parameters (Table 5). Moreover, VA correlated with mCSf and PWA (Table 6, Figure 3). 

## 4. Discussion

Our study confirmed that a chronic elevation in ICP induces biomechanical changes in the ONH, which can result in papilledema. The one-hour ICP lumbar monitoring demonstrated that dynamic changes in ICP correlate to structural optic disc damage in IIH patients, as shown in several studies [18,19,33,34,35]. More interestingly, we demonstrated that a higher PWA is associated with increased visual acuity loss and optic disk oedema.

Papilledema can be detected clinically by fundus examination even without pupil dilation; however, OCT allows the quantification of the extent of morphological alteration of the optic nerve associated with papilledema with a topographical analysis.

In our study, the RNFL thicknesses of the four sectors were all increased, especially in the inferior sector. Indeed, several studies have demonstrated the efficiency of OCT in identifying and measuring RNFL edema and that a significant increase in the mean RNFL thickness in all four quadrants is evident in eyes with papilledema [21,36,37,38].

As already reported [14,18], all measurements of ONH in patients with IIHWP were abnormal compared to IIHWOP. In particular, NRRT and NRRA were significantly increased, and optic CV was significantly reduced. IIHWP patients also showed significantly reduced CV and C/D. In another study, a short-term increase in CSF pressure associated with significant changes in ONH morphology was reported following the Valsalva maneuver. These findings suggested that ONH parameters may undergo sensitive changes in response to increased ICP and may also support our results [16]. 

Our study demonstrated that all ONH parameters are associated with CSF pressure monitoring data and visual acuity outcomes, presenting a correlation between edema severity and ICP in IIH, as corroborated in other studies [39]. Moreover, in line with our results, a positive correlation between the volumetric measurements of retinal surface elevation estimated from stereo fundus photographs and OCT and the severity of papilledema measured by grade of Frisén was reported [40,41,42]. Furthermore, papilledema can be regarded as a result of a compartmentation process involving the subarachnoid space of the optic nerve and causing, in the end, a toxic milieu around the nerve. For these reasons, several studies have proposed OCT parameters, such as the total retinal thickness and the peripapillary RNFL thickness, as useful markers for the quantification of papilledema severity [18,41].

It is well established that the optic nerve is under the influence of hydrostatic pressure along its length, and an alteration in this pressure may play a role in the etiology of papilledema [19,20]. Experimental studies designed to measure the eye lamina cribrosa pressure gradient have demonstrated a relationship between retrolaminar tissue pressure, lateral ventricle CSF pressure, and the optic nerve subarachnoid space [17]. It has been shown that CSF pressure influences retrolaminar tissue pressure; thus, CSF pressure plays a dominant role in the eye translaminar optic nerve tissue pressure gradient. Moreover, the existence of hydrostatic continuity between the lateral ventricle and optic nerve subarachnoid space has also been established [17,18]. 

More interestingly, PWA seems to strongly correlate with RNFL, CV, C/D ratio, and rim thickness. This finding, though limited by the small size of the patient population studied, could be interpreted assuming that PWA rises in conjunction with intraocular pulse amplitude; it is conceivable that an increase in both CSF and intraocular pressure may determine the elevation in the eye’s venous pressure and orbit-lowering venous blood flow in the retina, disc, and choroid, resulting in papilledema [43]. Our data suggest that the degree of PWA increase contributes more to neuroretinal tissue damage than the opening pressure level, inducing structural and metabolic alterations in the optic nerve in IIH patients. CSF PWA can be considered as a measure of the intracranial pressure pulsation strongly associated with the systolic and diastolic components of arterial pressure [44], thus representing a valuable CSF parameter to estimate intracranial compliance [31]. CSF PWA is a result of intracranial blood circulation and is strongly associated with the R-wave of an electrocardiogram [45]. Consequently, any cerebral volume alteration acting on the lateral ventricles, such as increased ICP or brain–water imbalance, potentially induce an increase in the CSF PWA [45]. Similarly, in IIH, the increased level of ICP may lead to the compression of cerebral bridging veins and, as a consequence, to a raised venous resistance to outflow [46]. Since it is closely related with the pressure–volume curve, an increase in the CSF PWA proceeds linearly with the intracranial CSF pressure. Indeed, as per the model of pressure–volume compensatory reserve, a decrease in intracranial compliance raises the CSF PWA linearly with the increment in ICP. Particularly, the rise in the CSF PWA curve from the flat to the exponential zone may represent an expression of the transition from good to poor compensatory reserve status [47]. To confirm this, abnormal levels of CSF PWA are usually tracked in patients with idiopathic normal pressure hydrocephalus, in which intracranial compliance is typically impaired [31,48]. 

This study has several limitations: the small sample size of our study and the absence of a control group may have limited the statistical power of our results. In addition, a physiological time-lapse existed between the OCT assessment and the ICP monitoring (approximately 24 h). Larger prospective studies are needed to confirm our findings. However, it should be noted that IIH being a rare disease made it difficult to find patients with the condition to perform the CSF monitoring; hence, the sample size may be considered adequate in such conditions. We also found that retinal measurements in the group of patients with papilledema were different between the right eye and left eye. There are several pieces of evidence that the severity of papilledema may be asymmetric, although it is uncommon [49,50]. The finding of worse papilledema in the right eye compared to left eye in some patients of our small cohort may have contributed to such differences. Moreover, in our study, patient groups showed differences in BMI and age. Although data on the correlation between BMI and OCT macular thickness are controversial [51], several studies have shown that BMI and OCT-measured retinal thickness are related [52,53]. Nevertheless, the effect of weight on peripapillary thicknesses is still poorly understood. Lastly, the cross-sectional design of this study did not allow us to define the prognostic value of the CSF dynamic parameters on ONH structure in IIH and to understand the differences in retinal and ONH profiles between IIHWP and IIHWOP. Thus, further studies with a longitudinal design are warranted.

## 5. Conclusions

Our data demonstrated that, during CSF short-term monitoring, the increased mean ICP and resulting mean PWA correlated with the OCT changes, indicating a reduced intracranial compliance. The combined assessment of OCT and CSF monitoring seemed to present reliable and sensitive measurements of elevated ICP in IIH. In particular, PWA, as an index of vascular dynamic changes, seemed to better correlate with all neuroretinal parameters, especially to C/DR, a mechanical and structural risk factor that leads to disc oedema in a self-perpetual cycle. The clinical value of PWA in monitoring the course of IIH needs to be evaluated in longitudinal studies.

## Figures and Tables

**Table 1:** Clinical and demographic characteristics.

	IIHWP (*n* = 9)	IIHWOP (*n* = 7)	*p* Value
Age (mean ± SD)	28.5 ± 7.9	31.9 ± 10.4	0.5
Female (%)	5(55.5)	4 (57.14)	-
BMI (mean ± SD)	40.5 ± 4.3	32.9 ± 3.7	0.002
Visual acuity (LogMAR) (mean ± SD) OD	0.53 ± 0.21	0.061 ± 0.02	0.001
OS	0.51 ± 0.18	0.058 ± 0.03	0.001
Intraocular pressure (mmhg)			
(mean ± SD)			
OD	15.2 ± 2	13.3 ± 1.6	0.07
OS	13.7 ± 3.7	14.9 ± 4	0.08

IIH: idiopathic intracranial hypertension; IIHWP: IIH with papilledema; IIHWOP: IIH without papilledema; BMI: body mass index; LogMAR: logarithm of minimal angle of resolution; OD: right eye; OS: left eye.

**Table 2:** CSF pressure monitoring data ^a^ in IIH with and without papilledema.

	IIHWP	IIHWOP	*p* Value
oCSFp (mmH_2_O)	348.1 ± 50.4	222.2 ± 14.7	0.0001
mCSFp (mmH_2_O)	370.6 ± 51.4	251.6 ± 21.4	0.0001
CSF PWA (mmH_2_O)	171.2 ± 13.1	62.3 ± 13.2	0.0001

^a^ data are given as mean ± SD. CSF: cerebrospinal fluid; IIH: idiopathic intracranial hypertension; IIHWP: IIH with papilledema; IIHWOP: IIH without papilledema; oCSFp: opening cerebrospinal fluid pressure; mCSFp: mean cerebrospinal fluid pressure; CSF PWA: cerebrospinal fluid pressure pulse wave amplitude.

**Table 3:** Retinal characteristics ^a^ in IIH with and without papilledema.

	IIHWP	IIHWOP	*p* Value
	(*n* = 9)	(*n* = 7)	
RNFL (µm)			
OD	209.4 ± 88.8	98.5 ± 5.6	0.005
OS	166.6 ± 54.5	98.6 ± 2.3	0.006
Superior sector (µm)			
OD	210.7 ± 95.0	119.6 ± 8.0	0.03
OS	177.2 ± 71.9	117.9 ± 7.2	0.05
Inferior sector (µm)			
OD	234.2 ± 65.4	120.0 ± 9.9	0.01
OS	184.9 ± 53.9	123.3 ± 6.6	0.01
Nasal sector (µm)			
OD	140.2 ± 64.3	81.7 ± 6.4	0.03
OS	127.1 ± 73.0	74.3 ± 6.7	0.05
Temporal sector (µm)			
OD	126.3 ± 36.8	69.7 ± 8.3	0.001
OS	113.0 ± 33.0	66.4 ± 6.5	0.003
Macular volume (µm)			
OD	10.2 ± 1.0	9.8 ± 0.4	0.3
OS	10.9 ± 1.1	9.9 ± 0.1	0.2
Foveal thickness (µm)			
OD	253.1 ± 15.0	242.6 ± 13.4	0.2
OS	268.8 ± 17.8	255.6 ± 12.3	0.1
Ganglion cell layer (µm)			
OD	80.0 ± 2.7	81.9 ± 4.8	0.3
OS	81.0 ± 3.5	82.6 ± 4.8	0.3
C/D ratio			
OD	0.01 ± 0.03	0.06 ± 0.04	0.01
OS	0.02 ± 0.01	0.05 ± 0.03	0.01
Rim thickness (µm)			
OD	757.2 ± 158.3	580.2 ± 127.4	0.03
OS	750.0 ± 142.5	577.8 ± 122.5	0.02
Rim area (µm^2^)			
OD	1.7 ± 0.2	1.4 ± 0.2	0.01
OS	1.7 ± 0.1	1.4 ± 0.3	0.01
Cup volume (µm^3^)			
OD	0.01 ± 0.04	0.07 ± 0.03	0.005
OS	0.02 ± 0.03	0.08 ± 0.01	0.005

^a^ data are given as mean ± SD. IIH: idiopathic intracranial hypertension; IIHWP: IIH with papilledema; IIHWOP IIH without papilledema; RNFL: retinal nerve fiber layer; OD: right eye; OS: left eye; C/D ratio: cap-to-disc ratio.

**Table 4:** Correlation analysis between retinal parameters and CSF pressure monitoring data.

	RNFL		C/D Ratio		Disc Area		Rim Thickness		Rim Area		Cup Volume	
	OD	OS	OD	OS	OD	OS	OD	OS	OD	OS	OD	OS
oCSFp	0.58 °	0.55 °	−0.59 °	−0.61 °	0.23	0.21	0.41	0.38	0.23	0.19	−0.64 *	−0.63 *
mCSFp	0.62 #	0.60 #	−0.55 °	−0.53 °	0.18	0.15	0.71 *	0.68 *	0.43	0.38	−0.55 °	−0.57 °
CSFPWA	0.78 *	0.72 *	−0.65 #	−0.63 #	0.17	0.16	0.72 *	0.71 *	0.41	0.40	−0.78 *	−0.72 *

The results are expressed as the Spearman rank correlation (r) and 95% confidence intervals (CIs). CSF: cerebrospinal fluid; OD: right eye; OS: left eye; RNFL: retinal nerve fiber layer; C/D ratio: cap-to-disc ratio; oCSFp: opening cerebrospinal fluid pressure; mCSFp: mean cerebrospinal fluid pressure, CSF PWA: cerebrospinal fluid pressure pulse wave amplitude. *: <0.001, #: <0.01, °: <0.05.

**Table 5:** Correlation analysis between retinal parameters and visual acuity data.

	RNFL		C/D Ratio		Disc Area		Rim Thickness		Rim Area		Cup Volume	
	OD	OS	OD	OS	OD	OS	OD	OS	OD	OS	OD	OS
VA OD	0.74 *	/	−0.60 #	/	0.19	/	0.33	/	0.21	/	−0.65 *	
VA OS	/	0.71 *	/	−0.61 #	/	0.20	/	0.31	/	0.17		−0.61 *

The results are expressed as the Spearman rank correlation (r). OD: right eye; OS: left eye; RNFL: retinal nerve fiber layer; C/D ratio: cap-to-disc ratio; VA: visual acuity expressed as logarithm of minimal angle of resolution (LogMAR). *: <0.001; #: <0.01.

**Table 6:** Correlation analysis between CSF pressure monitoring and visual acuity data.

	oCSFp	mCSFp	CSF PWA
VA OD	0.40	0.64 #	0.64 *
VA OS	0.42	0.62 #	0.66 *

The results are expressed as the Spearman rank correlation (r). OD: right eye; OS: left eye; VA: visual acuity expressed as logarithm of minimal angle of resolution (LogMAR); oCSFp: opening cerebrospinal fluid pressure; mCSFp: mean cerebrospinal fluid pressure; CSF PWA: cerebrospinal fluid pressure pulse wave amplitude. *: <0.001, #: <0.01.

## Data Availability

The datasets used and/or analyzed during the current study are available from the corresponding author on request.
